# PPARG is a potential target of Tanshinone IIA in prostate cancer treatment: a combination study of molecular docking and dynamic simulation based on transcriptomic bioinformatics

**DOI:** 10.1186/s40001-023-01477-w

**Published:** 2023-11-06

**Authors:** Tongtong Zhang, Xinglin Chen, Xiran Ju, Jixiang Yuan, Jielong Zhou, Zhihang Zhang, Guanqun Ju, Dongliang Xu

**Affiliations:** 1 Urology Centre, Shuguang Hospital Affiliated to Shanghai University of Traditional Chinese Medicine, Shanghai, 200000 China; 2 Institute of Surgery of Integrated Traditional Chinese and Western Medicine, Shuguang Hospital Affiliated to Shanghai University of Traditional Chinese Medicine, Shanghai, 200000 China

**Keywords:** Tanshinone IIA, Prostate cancer, Molecular docking, Molecular dynamics, Bioinformatics

## Abstract

Tanshinone IIA is a lipophilic organic compound from the root of Danshen (Salvia miltiorrhiza) and is one of the most well-known Tanshinone molecules by pharmacologists. In recent years, in addition to effects of anti-cardiovascular and neurological diseases, Tanshinone IIA has also shown some degrees of anti-prostate cancer potential. Although they do have some studies focusing on the molecular mechanism of Tanshinone IIA’s anti-prostate cancer effects, a further understanding on the transcriptomic and structural level is still lacking. In this study, transcriptomic sequencing technology and computer technology were employed to illustrate the effects of Tanshinone IIA on prostate cancer through bioinformatic analysis and molecular dynamics simulation, and PPARG was considered to be one of the targets for Tanshinone IIA according to docking scoring and dynamic calculation. Our study provides a novel direction to further understand the mechanism of the effects of Tanshinone IIA on prostate cancer, and further molecular biological studies need to be carried on to further investigate the molecular mechanism of Tanshinone IIA’s anti-prostate cancer effect through PPARG.

**Supplementary Information:**

The online version contains supplementary material available at 10.1186/s40001-023-01477-w.

## Introduction

Prostate cancer, which is one of the most common causes of cancer death in men, estimated new cases of nearly 1.4 million and induced 375,000 deaths around the world, second only to lung cancer [1]. In recent years, significant progress has been made in the early diagnosis and treatment of prostate cancer through widespread screening based on Prostate-Specific Antigen (PSA), the application of multiparametric magnetic resonance imaging (mpMRI), and the use of composite predictive indicators, such as the Prostate Health Index (PHI) [2–4]. Current clinical treatments for PCa includes active surveillance, surgical procedure, radiation therapy, androgen deprivation therapy (ADT) and chemotherapy [5]. For hormone sensitive PCa (HSPC), known as the early stage of PCa, ADT to inhibit the androgen receptor pathways is always the first-line treatment, but even until today, the clinical efficacy of ADT is still not satisfactory and results in a castration-resistant PCa which is the incurable stage of PCa [6]. Provided numerous side effects of ADT, such as obesity, osteoporosis and muscle loss, it is clinically necessary to develop alternative drugs to treat HSPC without hormonal disruption [7].

Given that a large majority of clinical cancer suppress drugs are known to be derived from herbs, great attention has been paid to natural products due to their valuable anti-tumor bioactivities and clinically translational potential [8]. Danshen, dried root of a famous Chinese herb, Salvia miltiorrhiza, is an important traditional medicine in China, which is expected to have variety of therapeutic effects on cardiovascular system [9]. Many bioactive compounds have been discovered in the extraction of Danshen, including cryptotanshinone, Tanshinone I, Tanshinone IIA and Tanshinone IIB. Among the above tanshinone molecules, Tanshinone IIA is the most abundant and the most investigated lipophilic tanshinone component [10]. Tanshinone IIA has been established to have diverse bioactive properties, including anti-oxidation [11], anti-apoptosis [12] and anti-inflammation [13] abilities. Recent studies focusing on its tumor suppression effects have also reported the anti-proliferative potential of Tanshinone IIA to HSPC through androgen receptor inhibition [14, 15], cell cycle arrest, p53 signaling activation, PI3K/AKT pathway inhibition and endoplasmic reticulum stress [16–18]. Chemical modified derivatives based on the structure of Tanshinone IIA have been effectively designed aiming at the treatment of prostate cancer [19, 20]. However, comprehensive investigations such as transcriptomic studies on the anti-HSPC mechanism of Tanshinone IIA are still lacking, which impeded the development of Tanshinone IIA derives and its potential clinical application.

In the present study, a most universally investigated HSPC cell line, LNCaP, was treated with Tanshinone IIA and a transcriptomic study was afterwards conducted. With Tanshinone IIA induced transcriptomic effects identified, bioinformatic analysis and molecular dynamics evaluation revealed the potential role of PPARγ (PPARG) in the anti-HSPC activity of Tanshinone IIA. Our study indicated a reasonable mechanism of Tanshinone IIA against HSPC and provided a theoretical basis for further utilization of this classic natural product.

## Methods

### Cell culture and reagents

Human androgen-dependent prostate cancer cell line LNCaP was obtained from American-Type Culture Collection (ATCC). LNCaP cells were cultured using Roswell Park Memorial Institute 1640 medium (RPMI-1640, Gibco) and supplemented with 10% Fetal Bovine Serum (FBS, Gibco) under the condition of 37 ℃ and 5% CO2. Tanshinone IIA (Macklin) was dissolved in dimethylsulfoxide (DMSO), adjusted to 2.5 mg/ml and store at -80℃.

### Cell viability assay and colony formation assay

According to the previous study conducted by other investigators, Tanshinone IIA has a half maximal inhibitory concentration (IC50) of 2.5 μg/ml to LNCaP cells within 24 h [16], and we followed the pre-existed IC50 to treat the LNCaP cells 24 h with a 2.5 μg/ml Tanshinone IIA in our subsequent studies. 2000 LNCaP cells were seeded in a 96 well cell culture plate under the condition of 37℃ and 5% CO2 for 24 h and cultured with 2.5 μg/ml Tanshinone IIA for another 48 h. Cell Counting Kit-8 (Beyotime) was used to analyze the cell viability according to the manufacturer’s protocol. For colony formation assay, 200 LNCaP cells were seeded in a 6 well cell culture plate for 24 h and cultured with 2.5 μg/ml Tanshinone IIA for 7 days. Cells were fixed with 4% formaldehyde and dyed with crystal violet to observe the colony formation. Data were presented as mean ± sd, and *t* test was applied to evaluate the statistical significance.

### Tanshinone IIA treatment and total RNA extraction

LNCaP cells were seeded in a 6 well cell culture plate with 2 ml medium in each well. 2.5 mg/ml DMSO dissolved Tanshinone IIA solution was diluted with RMPI-1640 medium to obtain a working Tanshinone IIA solution of a concentration of 2.5 μg/ml. Medium were changed into working solution at the time when the growth of LNCaP cells reached about 80% confluency and 0.1% DMSO was set as control. After 24 h, total RNA was harvest using commercial extraction kits (RNAeasy™ Plus, Beyotime) following the manufacturer’s instructions. Briefly, LNCaP cells were lysed and centrifuged to remove cell debris, and buffers of different functions were sequentially added, and total RNA was consequently dissolved in RNase-free water and stored at -80℃.

### RNA sequencing

For total RNA extraction, the concentration and integrity number of RNA were acquired applying Fragment Analyzer 5400 (Agilent, USA) for further process. mRNA was enriched and purified by oligo(dT)-modified magnetic beads and subsequently segmented by reagents under appropriate conditions. Single strand and double strand cDNA fragments were synthesized with poly-A tail added. After end repair and circularization, cDNA library was established as single strand DNA and then amplified into DNA nanoballs. Sequencing process was conducted using the platform of BGIseq500 (BGI-Shenzhen, China) to get single end 50 bases reads.

### Quality control and filtering

Raw reads were obtained and the quality control process was then performed applying the filtering platform SOAPnuke (BGI-Shenzhen, China). For reads filtering and quality control, we followed the below steps: 1) eliminate the reads with the adaptor contained (adaptor pollution); 2) exclude reads with an unknown base content ratio greater than 5%; and 3) eliminate reads with the proportion of bases with a quality score less than 15 greater than 20%. The filtered reads were then processed to bioinformatic analysis.

### Bioinformatic analysis and statistics

Differential gene expression profile was established with *q* < 0.05 and |Log2 fold change|≥ 1. Kyoto Encyclopedia of Genes and Genomes (KEGG, https://www.genome.jp/kegg/) and Gene Ontology (GO, http://geneontology.org/) enrichment was performed to investigate the biological significances of Tanshinone IIA induced differentially expressed gene profile, and the potentially interfered transcription factors were predicted using online website The Database for Annotation, Visualization and Integrated Discovery (DAVID, https://david.ncifcrf.gov/). The chemical structure of Tanshinone IIA (CAS: 568–72-9) was downloaded from PubChem (https://pubchem.ncbi.nlm.nih.gov/) and a pharmacophore-based target fishing online database Pharmmaper (http://www.lilab-ecust.cn/pharmmapper/) was referred to predict the Tanshinone IIA–protein interactions [21]. Gene–gene network was obtained from STRING database (https://string-db.org/) and analyzed by Cytoscape 3.8.2.

To further investigating the clinical significance of Tanshinone IIA-induced transcriptomic alteration, 553 transcriptomic data and the corresponding clinical information were obtained from The Cancer Genome Atlas (TCGA–PRAD, https://portal.gdc.cancer.gov/). R software 4.2.1 was applied for analysis and visualization. For Progress-Free Interval (PFI) analysis, we employed R pack survival [3.3.1], survminer, ggplot2[3.3.6] and Log-rank test; for expression difference analysis, we used R pack ggplot2[3.3.6], stats [4.2.1], car [3.1–0] and Kruskal–Wallis test (if not applicable, Mann–Whitney *U* test would be used). *P* < 0.05 was considered as statistical significantly.

### Molecular docking

AutoDock Vina 1.1.2 software was applied to calculate the binding mode of Tanshinone IIA to PPARA, PPARG and STAT1. The crystal structure of PPARA, PPARG and STAT1 were obtained from Protein Data Bank database (https://www.rcsb.org/), with the corresponding PDB ID 3ET1, 7AWD and 7NUF. The solvent and original ligands were removed, with the hydrogen atoms the atomic charges added. Tanshinone IIA structure obtained from PubChem database were energy minimized, atomic charges added to facilitate the docking process. For PPARA and STAT1, we set the grid box with the size of 30 × 30 × 30 Å^3^, including the existed active binding sites, and for STAT1, we predicted the molecular cavity using Discovery Studio Visualizer 20.1 as the active binding site. All the protein and chemical structures were converted into PDBQT format by AutoDock Tools 1.5.6 before molecular docking and the non-bond interactions were analyzed by Discovery Studio Visualizer 20.1.

### Molecular dynamics

The original structure of the Tanshinone IIA–protein complex was used as the starting point for conducting whole-atomic molecular dynamics simulations, employing AMBER v.18 [22]. Prior to the simulations, we obtained the charges of the small molecules using the Hartree–Fock (HF) SCF/6-31G* calculations performed using the software antechamber module and Gaussian 09 [23, 24]. Following the charge calculation, we described the small molecules and proteins using the GAFF2 small molecule force field and ff14SB protein force field, respectively [25, 26]. For each system, the LEaP module was utilized to add hydrogen atoms, and a truncated octahedral TIP3P solvent box was placed at a distance of 10 Å from the system [27]. Na + and Cl- ions were added to the system to balance the overall charge. Finally, topology and parameter files required for the simulations were generated.

Molecular dynamics simulations were performed using AMBER v.18 software [22]. Prior to the simulations, the system underwent energy optimization, which involved 2500 steps of steepest descent method followed by 2500 steps of conjugate gradient method. This optimization process aimed to minimize the energy of the system.

After the energy optimization, a 200 ps ramp-up simulation was conducted to gradually increase the system temperature from 0 K to 298.15 K at a fixed volume and a constant ramp-up rate. This step allowed the system to equilibrate slowly and reach the desired simulation temperature. Subsequently, a 500 ps NVT (constant number of particles, volume, and temperature) simulation was performed at 298.15 K to further distribute the solvent molecules uniformly within the solvent box.

Following the NVT simulation, an equilibrium simulation of 500 ps was carried out for the entire system under NPT (constant number of particles, pressure, and temperature) conditions. In addition, two composite systems were simulated for 50 ns each under NPT tethering with periodic boundary conditions.

During the simulations, a non-bond truncation distance of 10 Å was set. The Particle Mesh Ewald (PME) method was employed to calculate long-range electrostatic interactions [28]. The SHAKE method was utilized to constrain the bond lengths of hydrogen atoms [29]. Temperature control was achieved using the Langevin algorithm, with a collision frequency (γ) set to 2 ps^−1^. The system pressure was maintained at 1 atm, and the integration step was set to 2 fs. Trajectories were saved at 10 ps intervals for subsequent analysis [30].

### Molecular mechanics with generalized Born and surface area solvation (MM/GBSA) calculation

The binding-free energy of the protein–ligand complex for all systems was calculated based on the MM/GBSA method [31–33]. The present calculation is based on the molecular motion trajectory at 45–50 ns in the aforementioned molecular dynamics simulation, using the following equation:

1 $${\mathbf{\Delta G}}_{{{\mathbf{bind}}}} = \, {\mathbf{\Delta G}}_{{{\mathbf{complex}}}} {-} \, \left( {{\mathbf{\Delta G}}_{{{\mathbf{receptor}}}} + \, {\mathbf{\Delta G}}_{{{\mathbf{ligand}}}} } \right) \, = \, {\mathbf{\Delta E}}_{{{\mathbf{internal}}}} + \, {\mathbf{\Delta E}}_{{{\mathbf{VDW}}}} + \, {\mathbf{\Delta E}}_{{{\mathbf{elec}}}} + \, {\mathbf{\Delta G}}_{{{\mathbf{GB}}}} + \, {\mathbf{\Delta G}}_{{{\mathbf{SA}}}}$$

In Eq. (1), **ΔE**_**internal**_ represents the internal energy, **ΔE**_**VDW**_ represented van der Waals interaction and **ΔE**_**elec**_ represented electrostatic interaction. The internal energy included the bond energy (Ebond), angular energy (Eangle), and torsion energy (Etorsion); In this study, the solvation-free energy was determined by considering both the polar solvation-free energy (**ΔG**_**GB**_) and the nonpolar solvation-free energy (**ΔG**_**SA**_). The polar solvation-free energy, **ΔG**_**GB**_, was calculated applying the GB model (igb = 2) developed by Nguyen et al. [34]. On the other hand, the nonpolar solvation-free energy, **ΔG**_**SA**_, was determined based on the data of the surface tension (γ) and the solvent accessible surface area (SASA). The equation used to calculate **ΔG**_**SA**_ was **ΔG**_**SA**_ = 0.0072 × SASA [35]. It is worth noting that, in this study, we neglected the entropy variation due to the high computational resource requirements and the relatively low accuracy associated with including entropy calculations [31].

## Results

### The anti-proliferative effects of Tanshinone IIA

**Fig. 1:**
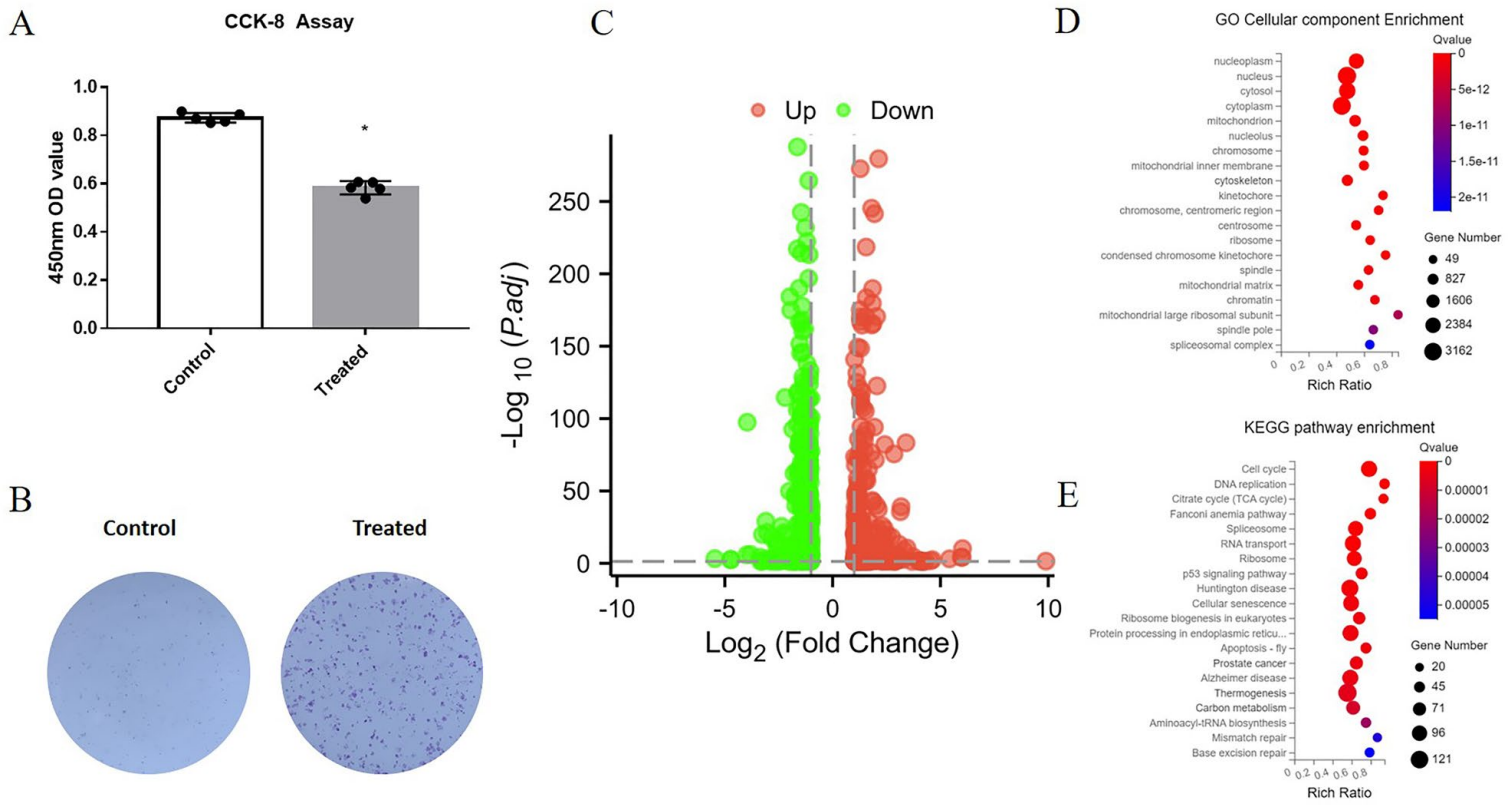
Anti-proliferation effects of Tanshinone IIA on LNCaP cells with transcriptomic analysis. **A** CCK-8 cell viability assay of LNCaP cells treated with 2.5 μg/ml Tanshinone IIA; **B** colony formation assay of LNCaP cells treated with 2.5 μg/ml Tanshinone IIA; **C** volcano plot of differentially expressed genes of LNCaP cells induced by 2.5 μg/ml Tanshinone IIA; **D** GO enrichment of differentially expressed genes; E. KEGG enrichment of LNCaP cells induced by 2.5 μg/ml Tanshinone IIA

The cell viability of LNCaP was significantly suppressed by 2.5 μg/ml Tanshinone IIA. Comparing the DMSO control group, the 450 nm OD value of LNCaP cells treated with Tanshinone IIA for 48 h decreased by about 0.32% and the colony formation ability was significantly suppressed by 2.5 μg/ml Tanshinone IIA, indicating that Tanshinone IIA may have a considerable anti-proliferative effect on LNCaP cells (Fig. 1A, B). The anti-proliferative effects were consistent with the previous study, further indicating the anti-HSPC potential of Tanshinone IIA [12, 16].

### Tanshinone IIA induced transcriptomic shift

After quality control and filtering, a significant transcriptomic change was induced by 2.5 μg/ml Tanshinone IIA, in which 339 genes were downregulated; meanwhile, 336 genes were upregulated applying the cutoff setting of *q* < 0.05 and |Log2 fold change|≥ 1 (Fig. 1C). GO enrichment revealed that these genes mainly expressed in cell, organelle and membrane, which have many molecular functions including binding, catalytic activity and transcription regulator activity and involved in biological processes, such as cellular process, metabolic process and biological regulation (Fig. 1D). KEGG pathway enrichment inferred the important roles these genes may participated in many important biomedical areas, such as human diseases, organismal systems, and genetic information processing (Fig. 1E).

### Tanshinone IIA induced transcriptomic features were associated with clinical information

**Fig. 2:**
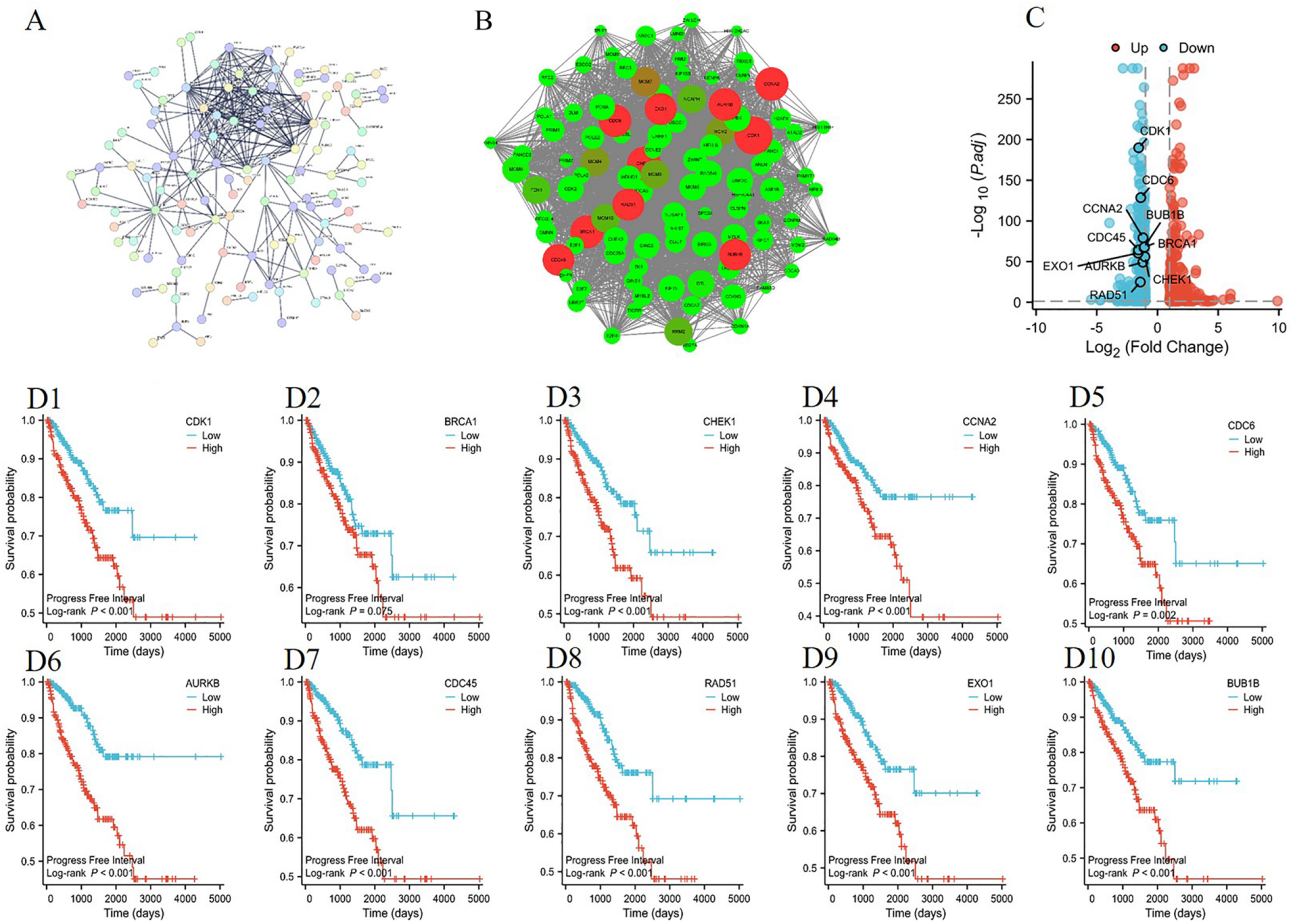
Tanshinone IIA related genes filtration with the prognostic potential evaluation. **A** PPI network imported from STRING; **B** hub genes selected by Cytoscape; **C** hub genes annotated on the volcano plot; **D1–D10**: Kaplan–Meier survival analysis of each hub gene

**Fig. 3:**
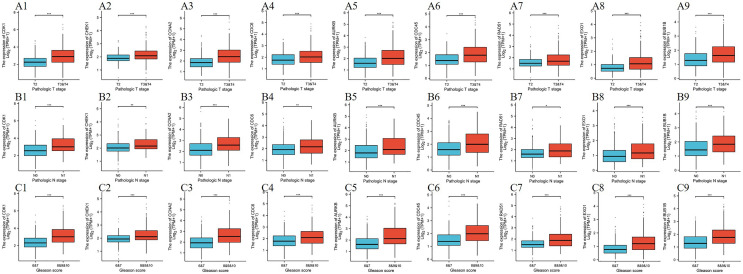
Pathological correlation of 9 Tanshinone IIA-associated key hub genes. **A1–A9:** the nine key hub genes are associated with pathological T stage; **B1–B9**: the nine key hub genes are associated with pathological N stage; **C1–C9**: the nine key hub genes are associated with pathological Gleason score

To investigate the relationship between Tanshinone IIA induced transcriptomic changes and clinical features of prostate cancer, we first obtained the gene–gene network from STRING database, and analysed these interactions using Cytoscape 3.8.2 (Fig. 2A). We selected the top 10 genes who had the highest degrees in the gene network as the distinctive Tanshinone IIA induced gene set (Fig. 2B). Surprisingly, the 10 selected genes were all down-regulated after Tanshinone IIA treatment (Fig. 2C), and the higher expression of all of which excepting BRCA1 were associated with a worse prognosis (shorter PFI) according to TCGA–PRAD data set (Fig. 2D1–D10). We further investigated the expression features of the remaining 9 genes (CDK1, CHEK1, CCNA2, CDC6, AURKB, EXO1, RAD51, CDC45 and BUB1B) in TCGA–PRAD, and found that upregulation of all the 9 genes were significantly associated with higher ISUP level (Gleason score, Fig. 3A1–A10), more lymph node metastasis (N, Fig. 3B1–B10) and heavier tumor load (T, Fig. 3C1–C10).

### Tanshinone IIA may interact with many transcription factors

It is well-known that RNA is synthesized by transcription factors according to the corresponding template DNA, so the functional alteration of transcription factor may be a causal of the transcriptomic changes. We uploaded the list of differentially expressed genes to DAVID online analytic system and obtained 126 potential functionally interfered transcription factors (Additional file 1). According to the chemical structure of Tanshinone IIA, Pharmmapper returned 248 target proteins which may interact with Tanshinone IIA (Additional file 2). We intersected the above 2 gene sets to get the transcription factors that Tanshinone IIA most possibly interacted with, and obtained 3 transcription factors which were PPARA, PPARG and STAT1 (Additional files 3 and 4).

### PPARG may have an affinity to Tanshinone IIA

**Fig. 4:**
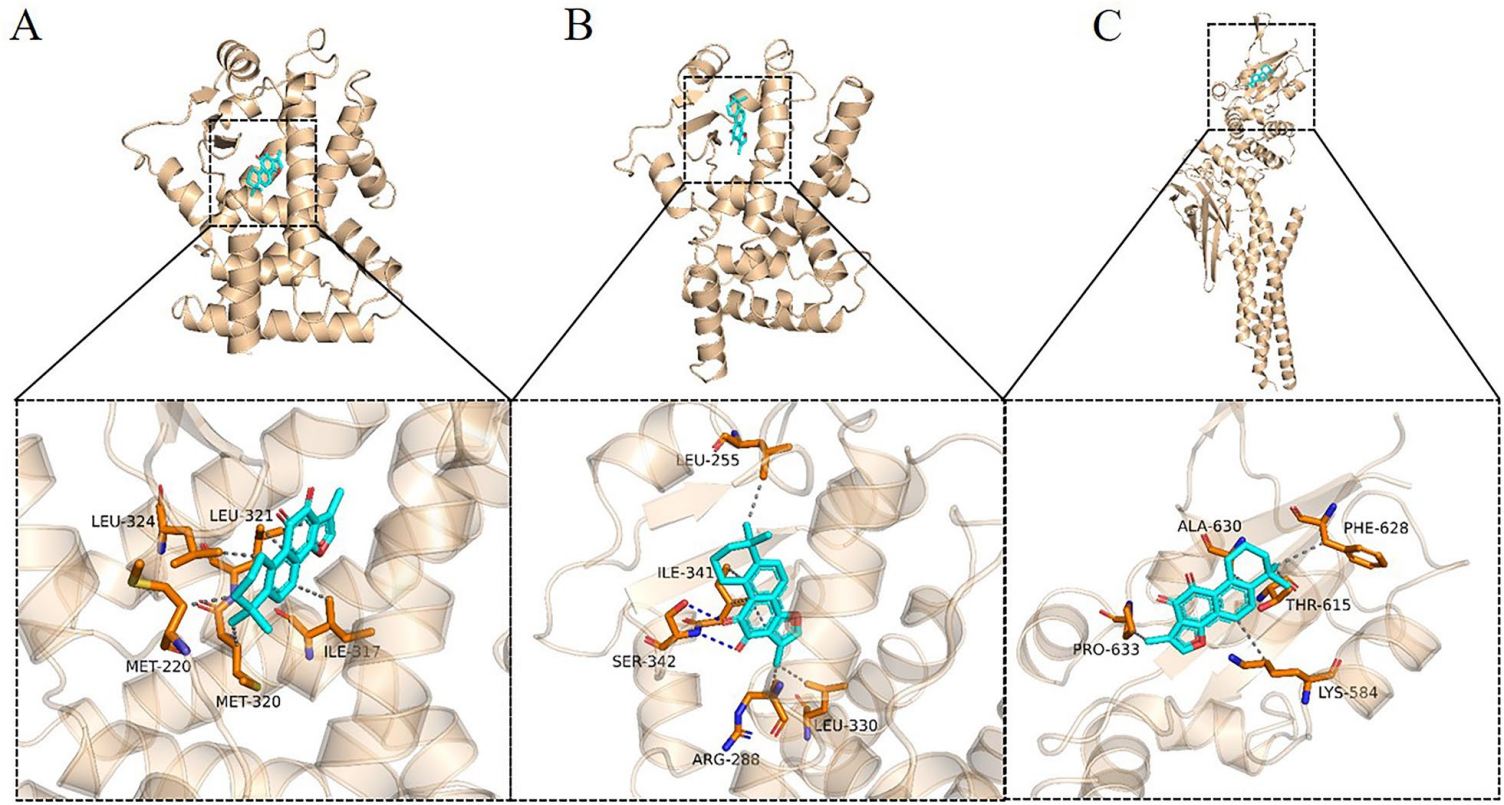
Diagram of Tanshinone IIA binding to protein macromolecules. Visualization was carried out based on the binding pattern of Tanshinone IIA and protein obtained by docking. The upper part is the overall view and the lower part is the local view. The cyan stick is small molecule; the orange cartoon is protein; the blue dotted line shows hydrogen bonding and the gray dotted line shows hydrophobic action. **A**: PPARA; **B**: PPARG; **C**: STAT1

To establish the potential transcription factor with the highest interaction possibility, we performed molecular docking with analysis of non-bond interactions of Tanshinone IIA to PPARA, PPARG and STAT1. The best binding modes were selected for non-bond interaction visualization (Fig. 4). The lowest binding-free energy of Tanshinone IIA to PPARA, PPARG and STAT1 was − 9.2, − 9.9 and − 7.0 kcal/mol, respectively, inferring PPARG might have the best binding affinity with Tanshinone IIA (Table 1). According to the non-bond interaction analysis, 1 Pi-Sulfur, 2 Pi–Pi T-shaped, 2 Alkyl and 1 Pi-Alkyl non-bond interactions were identified between Tanshinone IIA and PPARG, which may contribute to the decrease of binding energy. We also noticed that PPARG was predicted to regulate 461 genes in our transcriptomic study more than that of PPARA and STAT1, predicted to regulate 306 and 222 genes, respectively, which emphasized the importance of PPARG in the pharmacological effect of Tanshinone IIA (Additional file 1).

**Table 1:** Binding-free energies and energy components predicted by MM/GBSA (kcal/mol)

System name	PPARG_Tanshinone-IIA
ΔE_vdw_	− 43.08 ± 1.85
ΔE_elec_	− 0.25 ± 1.88
ΔG_GB_	13.33 ± 1.60
ΔG_SA_	− 5.10 ± 0.12
ΔG_bind_	− 35.10 ± 1.88

*ΔEvdW* van der Waals energy, *ΔEelec* electrostatic energy, *ΔGGB* electrostatic contribution to solvation, *ΔGSA* non-polar contribution to solvation, *ΔGbind* binding-free energy

### Molecular dynamic simulations of PPARG–Tanshinone IIA complex

**Fig. 5:**
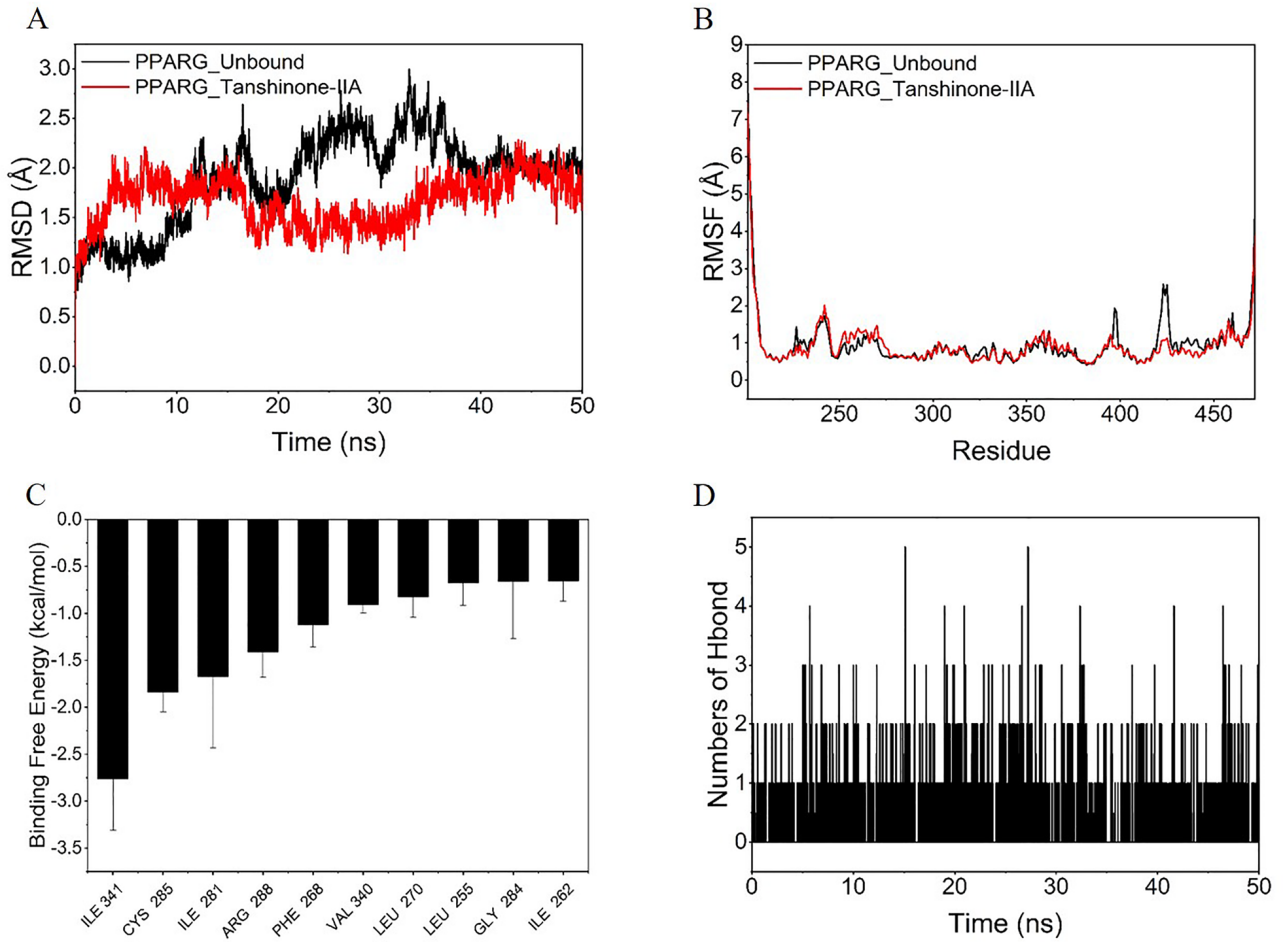
Molecular dynamic study of PPARG–Tanshinone IIA interaction. **A** Variation of the root mean square deviation (RMSD) plot of the systems during the 50 ns MD simulations; **B** root mean square fluctuation (RMSF) of the Cα atoms of PPARG systems in the 50 ns MD simulations; **C** Top 10 amino acid residues that contribute the most to the binding energy; **D** changes in the number of hydrogen bonds between small molecules and proteins during molecular dynamics simulations

The RMSD of molecular dynamics simulations can reflect the motion process of the complexes, and a larger RMSD as well as a more intense fluctuation indicates a violent motion, and vice versa, a smooth motion. According to the results of molecular docking above, we calculated the RMSD fluctuations of the PPARG empty protein and PPARG–Tanshinone IIA complex system during the simulation. As shown in Fig. 5A, the RMSD values of PPARG–Tanshinone IIA complex were lower than those of PPARG protein without binding the small molecule, and the fluctuations are smaller meanwhile, which indicated that the stability of PPARG–Tanshinone IIA complex was higher than that of PPARG protein without Tanshinone IIA binded, implying that Tanshinone IIA made the protein system movement stable and the binding effect of Tanshinone IIA to PPARG was significant.

RMSF can respond to the flexibility of proteins during molecular dynamics simulations. Usually, the binding of small molecules to a protein affects the intrinsic flexibility of the protein and thus the function of the protein. We calculated the RMSF of the empty protein PPARG as well as the protein after binding Tanshinone IIA. According to Fig. 5B, we can see that the RMSF of PPARG–Tanshinone IIA was much lower than that of the empty protein PPARG, implying that the small molecule Tanshinone IIA could stabilize the structure of PPARG and may possibly change the biological function of PPARG.

We estimated the binding energy applying the MM-GBSA method based on the trajectory of dynamic simulations, which can more accurately reflect the binding mode of small molecules to the target proteins. The calculation result showed that the binding energy of PPARG–Tanshinone IIA is −35.10 ± 1.88 kcal/mol. A negative value indicated that these two molecules have the potential to combined with each other automatically, with a lower value indicating a stronger binding. Obviously, our calculation showed that the binding affinity of PPARG–Tanshinone IIA was extremely strong. By energy decomposition, we could see that the main contributor to the binding of the complex was the van der Waals energy, followed by the non-polar solvation-free energy.

In addition, we calculated the binding-free energy contribution, and listed top-10 residues that contributed the most to the formation of PPARG–Tanshinone IIA complex. For PPARG–Tanshinone IIA, the top-10 contributing amino acid residues were ILE-341, CYS-285, ILE-281, ARG-288, PHE-268, VAL-340, LEU-270, LEU-255, GLY-284, and ILE-262 in order. The binding-free energy contribution of ILE-341 value was less than -2.5 kcal/mol, which was considered an important amino acid residue (Fig. 5C).

Hydrogen bonding is considered to be one of the strongest molecular interactions, and it is currently believed that a higher number of hydrogen bonds formed between small molecules and proteins indicates stronger interactions. In this study, we examined the changes in the number of hydrogen bonds formed during kinetic simulations. As shown in Fig. 5D, the number of hydrogen bonds formed by PPARG–Tanshinone IIA complex during the simulation fluctuated between 0 and 3 and remained at 0–1 hydrogen bonds most of the time, indicating that the contribution of hydrogen bonds to the complex formation is small, indicating that the binding energy of PPARG–Tanshinone IIA mainly originated from hydrophobic interactions.

## Discussion

Salvia miltiorrhiza (Danshen), a kind of famous traditional Chinese herb, is believed to treat diabetic angiopathy, organ fibrosis and Alzheimer's disease, and is widely and clinically applied by traditional Chinese medical care providers in the management of cardiovascular diseases, such as coronary artery disease [36–38]. Researchers have been extracting many promising chemical components, among which a series of lipophilic molecules named Tanshinone showed a great pharmacological potential [39]. Tanshinone IIA, a member of tanshinone family, is the most concerned Tanshinone compound in recent years because of its high content in Salvia miltiorrhiza [40]. Tanshinone IIA has been proved to be multi-functional which features the potential of inflammation suppressing, oxidative stress relieving and cardiovascular improving [39]. In addition to the effects on cardiovascular, endocrine and nervous systems, recently the anti-prostate cancer especially anti-HSPC potential of Tanshinone IIA has been revealed [16–18, 41–43]. Biomolecular mechanism of the HSPC suppression effects has been partially explained by investigators. Won, S. H. et, al. found that Tanshinone IIA can inhibit the proliferation and induce the apoptosis of LNCaP cell line (HSPC) by working as a PI3K/AKT pathway antagonist, which can suppress the surviving signals delivered by PI3K/AKT/mTOR pathway [17]. Tanshinone IIA exhibits cytotoxic effect on LNCaP cell line and LNCaP xenograft by induction of endoplasmic reticulum stress, p53 signaling activation and androgen receptor inhibition, and results in cellular cycle arrest at G0/G1 phase [16, 18]. However, there is still much to learn how Tanshinone IIA affects the viability of HSPC, and a comprehensive understanding based on transcriptomic level is in need for further exploitation of Tanshinone IIA.

In the present study, we established the anti-HSPC effect of Tanshinone IIA by CCK-8 cell viability assay after treating LNCaP cells with 2.5 μg/ml Tanshinone IIA solution, and then performed an mRNA sequencing to get a comprehensive understanding of Tanshinone IIA induced transcriptomic changes. According to the transcriptomic assay, we identified 339 downregulated genes and 336 upregulated genes with |Log2 fold change|≥ 1 and *p* value < 0.05. For further speculating the mechanism of transcriptomic change, we predicted the potential transcription factors of which the function interfered may result in the present transcriptomic changes using online predictor DAVID, and obtained 125 relative transcription factors. Applying pharmacophore-based target fishing system and molecular docking with dynamic simulation, we finally considered that Tanshinone IIA was most likely to interact with PPARG and lead to growth inhibition of LNCaP cells according to the affinity score and dynamic results. Few studies have demonstrated the effect of Tanshinone IIA on PPARG, which seems to be inconsistent [44, 45]. PPARG is well-known for its function in lipid metabolism and Tanshinone IIA can act as a PPARG antagonist with a dissociation constant of 2.562 ± 0.711 μM, and reduced body weight and blood lipid level [44]. PPARG plays an important role in biological behaviors of prostate cancer. PPARG is a classic transcription factor triggered by ligand, activating lipid signaling by upregulating acetyl-CoA carboxylase (ACC), fatty acid synthase (FASN) and ATP citrate lyase (ACLY), and promotes the metastatic prostate cancer [46]. PPARG overexpression also induces the upregulation of AKT3 and triggered mitochondrial ATP synthesis, contributing to prostate cancer progression with elevated energy supplies [47]. Previous study has considered the development of PPARG antagonist as anti-prostate cancer agent [48]. According to the binding potential of our results and the PPARG antagonistic effects of Tanshinone IIA in other study [44], Tanshinone IIA can probably acted as a PPARG antagonist in the inhibition of prostate cancer growth.

The advancement of computer science has significantly advanced the field of medical biology. Through the application of bioinformatics analysis and molecular dynamics simulations, we have successfully established a correlation between the pharmacological effects of Tanshinone IIA and the biological function of PPARG. While our research has yielded certain accomplishments, it is imperative to acknowledge certain limitations that necessitate further refinement. As an exploratory investigation, our study primarily relies on bioinformatics predictions and sequencing data, rendering our exploration at the molecular mechanism level somewhat superficial. Tanshinone IIA is a crucial natural product, and gaining insight into its mechanism of action is pivotal in facilitating further research and its potential application in the development of effective treatments for prostate cancer. However, based on previous research, prostate cancer can develop resistance to small molecule drugs through various mechanisms, such as the secretion of IGF-1 by adipocytes and the regulation of m6a methylation levels [49, 50]. As a small molecule drug, the potential resistance of prostate cancer to Tanshinone IIA should be a subject of concern in future research. To comprehensively elucidate the actual biological impact of Tanshinone IIA on PPARG, additional molecular biology experiments are required, thus enabling us to better understand the specific mechanism through which Tanshinone IIA combats prostate cancer. Considering that the Tanshinone IIA derivative, Tanshinone IIA Sodium Sulfonate, has been widely used in China without significant reported adverse reactions, there is a promising opportunity to incorporate Tanshinone IIA into clinical oncology efforts in the future [51].

## Conclusion

Our study applied transcriptomic sequencing and computer technology to suggest that Tanshinone IIA may suppress the proliferation of prostate cancer through interacting with PPARG, providing a promising direction for further understanding the anti-prostate cancer effects of Tanshinone IIA. Tanshinone IIA may work as a potential PPARG regulator in the progression of prostate cancer, which required further investigation.

### Supplementary Information

**Additional file 1.** Potential functionally interfered transcription factors predicted by DAVID.

**Additional file 2.** Tanshinone IIA targets predicted by Pharmmapper.

**Additional file 3.** Transcription factors predicted to be interfered with Tanshinone IIA.

**Additional file 4.** Venn diagram of transcription factors predicted to be interfered with Tanshinone IIA.

## Data Availability

All the data can be available from authors upon reasonable request.
