# Supplementary figures and images for: PPARG is a potential target of Tanshinone IIA in prostate cancer treatment: a combination study of molecular docking and dynamic simulation based on transcriptomic bioinformatics
Source: Eur J Med Res. 2023 Nov 6;28:487. doi: 10.1186/s40001-023-01477-w (PMC10626789; doi:10.1186/s40001-023-01477-w)

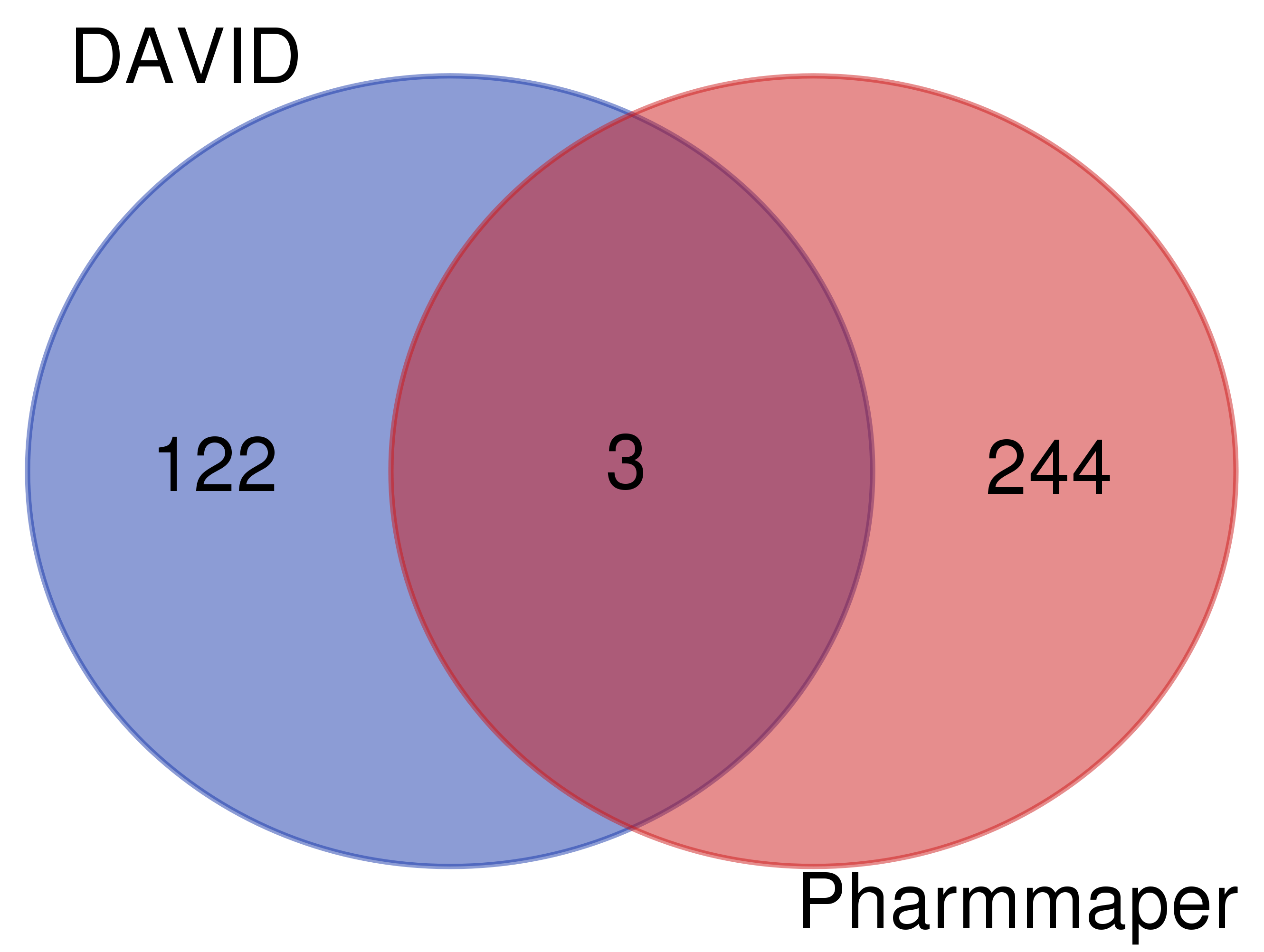

Supplement: Supplementary file 4 — Additional file 4. Venn diagram of transcription factors predicted to be interfered with Tanshinone IIA. [file 40001_2023_1477_MOESM4_ESM.png]
